# Augmenting pigmented lesion assay results with the three‐point dermoscopy checklist to improve pigmented lesion triage

**DOI:** 10.1111/srt.13205

**Published:** 2022-09-27

**Authors:** Joanna Ludzik, Alyssa L. Becker, Claudia Lee, Alexander Witkowski

**Affiliations:** ^1^ Department of Dermatology Oregon Health & Science University Portland Oregon USA; ^2^ John A. Burns School of Medicine University of Hawai'i at Manoa Honolulu Hawaii USA; ^3^ University of California Riverside School of Medicine Riverside California USA

## 1 INTRODUCTION

The estimated cost to the healthcare system to detect one melanoma is approximately $32594, which is largely due to the high biopsy rate of benign lesions.^1^ One systematic review and meta‐analysis found that the number needed to treat to identify one melanoma was 22.62 for primary care providers and 9.60 for dermatology providers.^2^ Pigmented lesion assays (PLA, “adhesive patch tests”), which are intended to be used on suspicious pigmented lesions that exhibit ≥1 ABCD criteria, noninvasively detect the presence of three genes associated with melanoma (LINC00518, PRAME, and TERT). PLAs have the potential to decrease the skin biopsy burden with a reported sensitivity of 91%–97% and negative predictive value (NPV) ≥99%.^3^, ^4^ Another tool used in pigmented skin lesion triage, the modified dermoscopy three‐point checklist (3PC), which includes asymmetry, round structures, and blue color, has been shown to have a sensitivity of 96.3% when employed by novice dermoscopists.^5^

## 2 METHODS

Our retrospective case–control study aimed to compare the sensitivities and NPVs of PLA‐testing and the 3PC in the context of suspicious pigmented lesions. We also investigated associations among PLA results, ABCD criteria, and 3PC features. Data was collected from April 2021 to August 2022 from an academic‐tertiary level center.

## 3 RESULTS

Of 510 total PLA cases, 67 were quantitatively insufficient, meaning that there was not enough genetic material to run the test, and 443 returned with a definitive result. Of those with a definitive PLA result, 117 were biopsied and 34 were melanoma (Table 1). Of the 34 biopsy‐proven melanomas, 12 were PLA− (Table 1). Based on our preliminary results, the PLA test's sensitivity and NPV for melanoma was 64.7% and 96.9%, respectively, when non‐biopsied clinically stable lesions were included as true negatives.

**TABLE 1:** Pigmented lesion assays (PLA) results of pigmented lesions that underwent biopsy

	All lesions that underwent PLA testing, excluding QNS	Lesions that underwent PLA‐testing and had associated clinical and dermoscopy photos
Melanoma (MM)		
PLA+ (% of MM)	22 (65%)	20 (65%)
PLA− (% of MM)	12 (35%)	11 (35%)
Total (% of all biopsied lesions)	34 (29.1%)	31 (29.8%)
Non‐melanoma skin cancer (NMSC)		
PLA+ (% of NMSC)	2 (100%)	2 (100%)
PLA− (% of NMSC)	0 (0%)	0 (0%)
Total (% of all biopsied lesions)	2 (1.7%)	2 (1.9%)
Atypical melanocytic nevi (AMN)		
PLA+ (% of AMN)	15 (43%)	15 (50%)
PLA− (% of AMN)	20 (57%)	15 (50%)
Total (% of all biopsied lesions)	35 (29.9%)	30 (28.9%)
Benign on biopsy		
PLA+ (% of benign)	21 (46%)	18 (44%)
PLA− (% of benign)	25 (54%)	23 (56%)
Total (% of all biopsied lesions)	46 (39.3%)	41 (39.4%)
All biopsied lesions		
PLA+ (% of all biopsied lesions)	60 (51.3%)	55 (52.9%)
PLA− (% of all biopsied lesions)	57 (48.7%)	49 (47.1%)
Total (% of all biopsied lesions)	117 (100%)	104 (100%)
Not biopsied, clinically stable		
PLA+ (% of not biopsied)	0 (0%)	0 (0%)
PLA− (% of not biopsied)	326 (100%)	289 (100%)
Total count (% of not biopsied)	326 (100%)	289 (100%)
Total		
PLA+ (% of all lesions)	60 (13.5%)	55 (14.0%)
PLA− (% of all lesions)	383 (86.5%)	338 (86.0%)
Total count (% of all lesions)	443 (100%)	393 (100%)

Overall, 393 of the 443 PLA cases with definitive results and 104 of the 117 biopsied cases had associated clinical and dermoscopy images (Table 1). Of the 31 melanomas, all exhibited ≥1 3PC feature (Table 2). The sensitivity and NPV of the dermoscopy 3PC for melanoma were both 100% when non‐biopsied clinically stable lesions were included as true negatives.

**TABLE 2:** ABCD and dermoscopy three‐point checklist criteria of biopsied lesions with pigmented lesion assays (PLA) results

	PLA result	≥1 ABCD criteria	Clinical asymmetry	Irregular borders	Multiple colors	Diameter ≥6 mm	≥1 dermoscopy three‐point checklist criteria	Asymmetry on dermoscopy	Round structures	Blue structures
MMs										
	PLA+ (*n* = 20)	95% (*n* = 19)	75% (*n* = 15)	70% (*n* = 14)	75% (*n* = 15)	80% (*n* = 16)	100% (*n* = 20)	95% (*n* = 19)	50% (*n* = 10)	30% (*n* = 6)
	PLA− (*n* = 11)	91% (*n* = 10)	55% (*n* = 6)	45% (*n* = 5)	64% (*n* = 7)	64% (*n* = 7)	100% (*n* = 11)	82% (*n* = 9)	9% (*n* = 1)	36% (*n* = 4)
	All MMs (*n* = 31)	94% (*n* = 29)	68% (*n* = 21)	61% (*n* = 19)	71% (*n* = 22)	74% (*n* = 23)	100% (*n* = 31)	90% (*n* = 28)	35% (*n* = 11)	32% (*n* = 10)
AMNs										
	PLA+ (*n* = 14)	87% (*n* = 13)	60% (*n* = 9)	67% (*n* = 10)	67% (*n* = 10)	60% (*n* = 9)	100% (*n* = 15)	100% (*n* = 15)	20% (*n* = 3)	20% (*n* = 3)
	PLA− (*n* = 16)	73% (*n* = 11)	47% (*n* = 7)	33% (*n* = 5)	53% (*n* = 8)	47% (*n* = 7)	87% (*n* = 13)	73% (*n* = 11)	33% (*n* = 5)	20% (*n* = 3)
	All AMNs (*n* = 30)	80% (*n* = 24)	53% (*n* = 16)	50% (*n* = 15)	60% (*n* = 18)	93% (*n* = 28)	93% (*n* = 28)	87% (*n* = 26)	27% (*n* = 8)	20% (*n* = 6)
Benign										
	PLA+ (*n* = 18)	61% (*n* = 11)	44% (*n* = 8)	33% (*n* = 6)	33% (*n* = 6)	56% (*n* = 10)	100% (*n* = 18)	89% (*n* = 16)	39% (*n* = 7)	28% (*n* = 5)
	PLA− (*n* = 23)	91% (*n* = 21)	43% (*n* = 10)	26% (*n* = 6)	43% (*n* = 10)	61% (*n* = 14)	96% (*n* = 22)	91% (*n* = 21)	39% (*n* = 9)	26% (*n* = 6)
	All benign (*n* = 41)	78% (*n* = 32)	44% (*n* = 18)	29% (*n* = 12)	39% (*n* = 16)	59% (*n* = 24)	98% (*n* = 40)	90% (*n* = 37)	39% (*n* = 16)	27% (*n* = 11)
Not biopsied, clinically stable										
	PLA− (*n* = 289)	79.9% (*n* = 231)	49.1% (*n* = 142)	48.4% (*n* = 140)	46.0% (*n* = 133)	52.6% (*n* = 152)	95.5% (*n* = 276)	87.2% (*n* = 252)	33.2% (*n* = 96)	11.1% (*n* = 32)

Abbreviations: AMN, atypical melanocytic nevi; MM, melanoma.

Significantly, only 9% of PLA− melanomas exhibited round structures on dermoscopy, whereas 50% of PLA+ melanomas included round structures (*p* = 0.023) (Table 2). There was no significant difference in ABCD criteria or other 3PC criteria between PLA+ and PLA− melanomas.

## 4 DISCUSSION

Those who are nonexperts in skin cancer detection may find themselves over‐relying on PLA results, which can lead to delayed diagnosis of provider‐selected skin lesions of concern, given that based on our preliminary results, the PLA test's sensitivity for melanoma was 26.3%–32.3% lower than previously reported values.^3^, ^4^ The PLA test was negative in 35% of biopsy‐proven melanomas and over half of AMNs. These lesions would be mismanaged if the provider only took into account the PLA result. Fortunately, the dermoscopy 3PC can easily be employed even by nonexpert dermoscopy users and serve as an additional screening tool when monitoring lesions that return PLA−, especially in lesions that lack round structures in dermoscopy. Our study's results confirmed the high sensitivity of the dermoscopy 3PC, which has been previously reported.^5^ Pairing PLA testing with simple dermoscopy criteria such as the 3PC can improve pigmented lesion triage and monitoring, even by nonexperts.

The external validity of this study is limited by the small sample size of biopsied lesions and variability among providers reading clinical and dermoscopy images. Future studies are required to explore the utility of more specific dermoscopy features being used for the selection of lesions appropriate for PLA‐testing and monitoring of suspicious lesions with negative PLA results. Additionally, identifying dermoscopy features that may be associated with LINC00518, PRAME, and TERT positivity may help better select lesions for testing.

## CONFLICTS OF INTEREST

The authors declare that there is no conflict of interest that could be perceived as prejudicing the impartiality of the research reported.

## Data Availability

The data that support the findings of this study are available from the corresponding author upon reasonable request.

## References

[1] Matsumoto M , Secrest A , Anderson A , et al. Estimating the cost of skin cancer detection by dermatology providers in a large healthcare system. J Am Acad Dermatol. 2018;78(4):701‐709.e1. 10.1016/j.jaad.2017.11.033 29180093 PMC5963718

[2] Petty AJ , Ackerson B , Garza R , et al. Meta‐analysis of number needed to treat for diagnosis of melanoma by clinical setting. J Am Acad Dermatol. 2020;82(5):1158‐1165. 10.1016/j.jaad.2019.12.063 31931085 PMC7167347

[3] Ferris LK , Moy RL , Gerami P , et al. Noninvasive analysis of high‐risk driver mutations and gene expression profiles in primary cutaneous melanoma. J Invest Dermatol. 2019;139(5):1127‐1134. doi:10.1016/j.jid.2018.10.041 30500343

[4] Gerami P , Yao Z , Polsky D , et al. Development and validation of a noninvasive 2‐gene molecular assay for cutaneous melanoma. J Am Acad Dermatol. 2017;76(1):114‐120.e2. doi:10.1016/j.jaad.2016.07.038 27707590 PMC5599145

[5] Soyer HP , Argenziano G , Zalaudek I , et al. Three‐point checklist of dermoscopy. DRM. 2004;208(1):27‐31. doi:10.1159/000075042 14730233

