# Correction to “Augmenting pigmented lesion assay results with the three‐point dermoscopy checklist to improve pigmented lesion triage.”

**DOI:** 10.1111/srt.70307

**Published:** 2026-02-10

J. Ludzik, A. L. Becker, C. Lee, A. Witkowski, “Augmenting Pigmented Lesion Assay Results With the Three‑Point Dermoscopy Checklist to Improve Pigmented Lesion Triage,” *Skin Research and Technology* 28, no. 6 2022: 877–879, https://doi.org/10.1111/srt.13205

In the published version, the institutional affiliation of author Joanna Łudzik was incomplete. Only the Oregon Health & Science University affiliation was listed. The correct affiliations should read:

1. Oregon Health & Science University, Department of Dermatology, Portland, USA

2. Department of Bioinformatics and Telemedicine, Jagiellonian University Medical College, Kraków, Poland.

We apologize for this error.

